# The Implementation of a Multi-institutional Multidisciplinary Simulation-based Resuscitation Skills Training Curriculum

**DOI:** 10.7759/cureus.3593

**Published:** 2018-11-14

**Authors:** Timothy Chaplin, Rylan Egan, Nicholas Cofie, Jeffrey JJ Gu, Tamara McColl, Brent Thoma

**Affiliations:** 1 Emergency Medicine, Queen's University, Kingston, CAN; 2 Medical Education and Simulation, Queen's University, Kingston, CAN; 3 Miscellaneous, Queen's University, Kingston, CAN; 4 Surgery, University of Saskatchewan, Saskatoon, CAN; 5 Emergency Medicine, University of Manitoba, Winnipeg, CAN; 6 Emergency Medicine, University of Saskatchewan, Saskatoon, CAN

**Keywords:** simulation, resuscitation, medical education, curriculum development

## Abstract

Competency-based curricula require the development of novel simulation-based programs focused on the assessment of entrustable professional activities. The design and delivery of simulation-based programs are labor-intensive and expensive. Furthermore, they are often developed by individual programs and are rarely shared between institutions, resulting in duplicate efforts and the inefficient use of resources.

The purpose of this study is to demonstrate the feasibility of implementing a previously developed simulation-based curriculum at a second institution. We sought to demonstrate comparable program-level outcomes between our two study sites.

A multi-disciplinary, simulation-based, resuscitation skills training curriculum developed at Queen’s University was implemented at the University of Saskatchewan. Standardized simulation cases, assessment tools, and program evaluation instruments were used at both institutions.

Across both sites, 87 first-year postgraduate medical trainees from 14 different residency programs participated in the course and the related research. A total of 226 simulated cases were completed in over 80 sessions. Program evaluation data demonstrated that the instructor experience and learner experience were consistent between sites. The average confidence score (on a 5-point scale) across sites for resuscitating acutely ill patients was 3.14 before the course and 4.23 (*p* < 0.001) after the course.

We have described the successful implementation of a previously developed simulation-based resuscitation curriculum at a second institution. With the growing need for competency-based instructional methods and assessment tools, we believe that programs will benefit from standardizing and sharing simulation resources rather than developing curricula de novo.

## Introduction

Postgraduate medical education programs in Canada and the United States are transitioning or have transitioned to competency-based medical education (CBME) models that incorporate national milestones and entrustable professional activities (EPAs) [1-2]. One of the challenges of implementing CBME is that it requires direct observation and demonstration of competence of stage-specific EPAs before progressing in training. Resuscitation-based EPAs are particularly difficult to assess because of their unpredictable occurrence and resulting lack of reliable direct observation [3-4]. Simulation can assist with these challenges by providing safe and reproducible experiences while also allowing expert observation, focused feedback, and deliberate practice [5]. Unfortunately, the development of simulation curricula requires considerable resources and many programs have a limited number of faculty with formal simulation training. Moreover, simulation curricula are traditionally developed at the individual residency program level and are rarely shared within or between teaching institutions.

The emerging number of online sources of free open access medical education material and published simulation cases is slowly targeting this problem. There are numerous websites and repositories that provide access to cases that have been used elsewhere and, in some instances, even peer-reviewed (MedEdportal.org, EMSimcases.com). However, these cases are rarely part of a comprehensive curriculum, often do not contain assessment tools linking them to specific EPAs or milestones, and frequently lack program evaluation instruments.

The objective of this report is to demonstrate the feasibility of adopting a simulation curriculum, including well-designed peer-reviewed cases, competency-based assessment tools tied to EPAs, and program evaluation instruments, between two institutions and multiple residency programs while demonstrating comparable program level outcomes.

## Materials and methods

The “Nightmares Course” at Queen’s University is a resuscitation skill training program that has been previously described [6]. The Nightmares program facilitates the challenging transition that all medical trainees encounter as they progress from medical students to postgraduate trainees with independent patient-care responsibilities [7]. The initial approach to a critically ill patient is a common source of anxiety at this stage of training and carries significant risk for the patient if not managed competently [8]. The curriculum is designed specifically to teach and assess the following EPA: “Recognizes an acutely unwell floor patient, calls for appropriate help and initiates a basic assessment and management plan.” The University of Saskatchewan sought to create a similar curriculum and contacted educators at Queen’s University. In a discussion between the two sites, it was determined that not only could the University of Saskatchewan adopt the Queen’s University curriculum, but that the same assessment and program evaluation instruments could be used across both sites. This study was approved by the Research Ethics Boards at Queen’s University and the University of Saskatchewan.

Settings and participants

Course participants in the 2017-2018 academic year included first-year postgraduate medical trainees who were responsible for in-patient call at the University of Saskatchewan and Queen’s University. Participation was mandatory and designated as protected academic time by all programs involved. Written consent was requested in order to use assessment data for research purposes.

Curricular design

In keeping with adult learning theory and principles of assessment within CBME, the curriculum utilized small group learning, spaced repetition, direct observation with immediate formative feedback, and assessment tools anchored to the course EPA [9].

Scenarios for the course were chosen based on the most common calls to the Kingston Health Sciences Centre’s Rapid Assessment of Critical Events Team for acutely unwell patients (Table 1). The trainees were instructed to behave as they would if they were the first physician called to assess an acutely unwell patient in the ward at night. Consultants and/or senior trainees were available on the phone when requested.

**Table 1:** Simulation Scenarios

1	Acute pulmonary edema
2	Pneumonia
3	Pulmonary embolism
4	Bradycardia
5	Hypertensive emergency
6	Seizure
7	Ventricular tachycardia
8	Hyperkalemia
9	Sepsis
10	Acute myocardial infarction
11	Anaphylaxis
12	Opiate intoxication

Simulation sessions took place between August and December 2017. Over this period, trainees participated in one session every four weeks in groups of four to six. Each simulation session was 90-180 minutes long and involved three or four simulated scenarios. Scenarios lasted for 10-15 minutes and took place in a simulation suite using a high-fidelity mannequin that was set up and operated by a simulation technician. A nurse acted as a confederate in the simulation suite and one or two faculty facilitators observed the scenario and then provided formative feedback during a semi-structured debrief.

Assessment of trainees

The “Nightmares Course” assessment of trainees was modified for implementation at both sites [6]. Trainees were assessed in several ways using the same terminology and entrustment scoring tools (Appendix 1). Before the start of the course, each trainee completed a general confidence score related to managing an acutely unwell patient (Appendix 2). The same score was repeated upon completion of the resuscitation curriculum.

Trainees were assessed during the course in a multisource fashion. After each simulated scenario, the leader was assessed by their peers, a nursing staff, and the attending physician(s) (Appendix 1). The leader also completed a scenario-specific self-entrustment assessment upon reading the case “stem” (Appendix 3) and again after leading the simulated case. The whole assessment was designed as a single five-point entrustment score [10] that was anchored to the course EPA, as well as a box with the heading “please explain your rating” for qualitative comments.

Institutional-specific methods: University of Saskatchewan

Scenarios were delivered at two sites - Saskatoon and Regina, depending on the location of each trainee. All sessions were conducted using a high-fidelity simulation mannequin, but the model differed between Saskatoon (SimMan 3G, Laerdal, Toronto, Canada) and Regina (Hal S3000 Tetherless Patient Simulator, Gaumard, Florida, USA). Twenty-three faculty credentialed in Anesthesia, Emergency Medicine, General Surgery, Internal Medicine, and Intensive Care Medicine were trained to conduct the simulation sessions. All facilitators were required to complete a simulation and debriefing course as part of taking on this role.

Institutional-specific methods: Queen’s University

All scenarios were delivered at Queen’s University using a high-fidelity simulation mannequin (SimMan Essential, Laerdal, Toronto, Canada). A group of 10 faculty facilitators credentialed in Anesthesia, Emergency Medicine, General Surgery, Intensive Care Medicine, and Internal Medicine conducted the simulation sessions and completed the entrustment scores for each scenario. All facilitators had a self-identified interest in simulation-based teaching and resuscitation.

Program evaluation

Anonymous feedback from trainees was gathered using a modified version of a previously validated tool: the Evaluation of Technology-Enhanced Learning Materials: Learner Perceptions (ETELM-LP) inventory. Basic demographic data were collected with this survey including age, residency training program, and previous simulation experience. Faculty completed a modified version of the Evaluation of Technology-Enhanced Learning Materials: Instructor Perceptions (ETELM-IP) inventory. Basic demographic data, including age, sex, and specialty area, were collected along with the program evaluation. For both program evaluation surveys, modifications included the removal of questions that were not relevant to the course, altering the wording of questions to better fit the course, and adding additional questions related to the course objectives.

## Results

Participant demographics

A total of 87 first-year postgraduate medical trainees from 14 different training programs participated in the resuscitation curriculum and provided written consent for their assessment data to be used for research purposes. Thirty-four trainees were from the University of Saskatchewan and 53 from Queen’s University (Table 2).

**Table 2:** Participant Demographics

		Queen’s University	University of Saskatchewan
Gender	Male	29	23
	Female	21	11
	Other	0	0
Age	<25	14	16
	26-30	33	14
	31-35	3	3
	>36	0	1
Residency Program	Anatomic Pathology	2	0
	Anesthesia	4	5
	Diagnostic Radiology	3	0
	Emergency Medicine	0	2
	General Surgery	4	4
	Internal Medicine	23	23
	Neurology	2	0
	Ophthalmology	2	0
	Orthopedic Surgery	3	0
	Obstetrics and Gynecology	3	0
	Physiatry	2	0
	Psychiatry	4	0
	Radiation Oncology	2	0
	Urology	1	0
Total		53	34

Assessment of trainees

A total of 226 cases were conducted across both sites, 93 at Queen’s University and 133 at the University of Saskatchewan. Fifty-nine of 87 trainees (68%) led a scenario and received feedback on their performance. Following a scenario, the leader received an average of 7.5 assessments: one from each of faculty and nursing staff, a self-assessment, and three to five peer assessments. Qualitative comments were included on 84% of assessments.

Program evaluation

Thirty-three of 34 trainees at the University of Saskatchewan, and 47 of 53 at Queen’s University completed the ETELM-LP (Table 3). A clerical error resulted in the deletion of three questions from the survey at Queen's University; these correspond to the blank rows in Table 3. Agreement with statements was ranked using a 7-point Likert scale, ranging from strongly disagree (1) to strongly agree (7). Notably, the following statements received average scores of 6.2, 6.0, and 6.0, respectively: “Course objectives were relevant to my needs,” “I received adequate feedback on my learning progress,” and “Overall, the course improved my ability to assess and manage the acutely unwell floor patient.” Across both sites, the average confidence score before the course was 3.14, following the course, this was 4.23 (*p* < 0.001). Institution-specific pre-course confidence scores for the University of Saskatchewan and Queen’s University were 3.00 and 3.26, respectively, and post-course confidence scores were 4.12 and 4.33, respectively (*p* < 0.001 for both comparisons).

**Table 3:** Evaluation of Technology-enhanced Learning Materials: Learner Perceptions Agreement ranked on a 7-point Likert scale

	Queen’s University	University of Saskatchewan
Instructions provided a good introduction to the course.	6.0	6.1
Course objectives, expectations, and policies were clearly stated.	5.7	5.9
The course was well organized.	6.3	6.4
Course objectives were relevant to my needs.	6.2	6.2
The course technologies supported the learning objectives.	6.4	6.3
The educational activities promoted the achievement of the course objectives.	6.2	6.2
There was a strong instructor presence/personal touch in the course.		6.1
Educational activities encouraged interaction and collaboration with other participants.	6.3	6.3
Requirements for interaction and collaboration with other participants were clearly articulated.	5.7	6.2
Face-to-face activities contributed meaningfully toward achieving the course learning objectives.	6.3	6.2
Assessments were appropriate for the course objectives, content, and activities.	6.0	5.8
I had sufficient opportunity to assess and reflect upon my learning progress.	5.7	5.9
I received adequate feedback on my learning progress.	5.1	6.0
I had sufficient opportunity to evaluate/provide feedback on the course.	6.0	6.2
I received adequate support for any technical issues encountered during this course.	5.7	5.9
I received adequate support for any questions or concerns that I had about my learning.	5.9	6.0
This course will change my practice.	6.1	6.0
Providing feedback to my peers helped me to enhance my own performance.	5.8	5.5
Receiving feedback from my peers helped me to enhance my own performance	5.6	5.7
Receiving feedback from the nurse helped me to enhance my own performance		5.7
Receiving feedback from the attending helped me to enhance my own performance		6.3
Assessing my own performance helped me to enhance my performance.	5.8	5.7
I was satisfied with the length of time spent on the course.	5.7	5.7
I was satisfied with the overall effectiveness of the instructors.	6.4	6.2
I think my performance in the simulation lab is representative of my clinical ability in the ‘real world’.	5.3	5.2
I was satisfied with the overall quality of the course.	6.2	6.2
I would recommend this course to other PGY-1 residents.	6.6	6.2
Overall, the course improved my ability to assess and manage the acutely unwell floor patient.	6.5	6.0

Twenty-two of 23 faculties at the University of Saskatchewan and 11 of 11 at Queen’s University completed the ETELM-IP (Table 4). Notably, using a similar 7-point Likert scale, the following statements received average scores of 6.3, 6.5, and 5.9, respectively: “the educational activities encouraged participants' engagement with course content,” “I was satisfied with the overall quality of the course,” and “I provided adequate feedback on residents’ learning progress.”

**Table 4:** Evaluation of Technology-enhanced Learning Materials: Instructor Perceptions Agreement ranked on a 7-point Likert scale

	Queen’s University	University of Saskatchewan
Instructions provided a good introduction to the course	6.2	6.0
Course objectives were relevant to participant needs.	6.5	6.3
The course technologies supported the learning objectives.	6.5	6.4
The educational activities encouraged participants' engagement with course materials / content.	6.4	6.3
The educational activities promoted participants' achievement of the course objectives.	6.3	6.2
I was able to contribute a personal presence / personal touch during the course’s delivery.	6.4	6.3
Educational activities encouraged participants' interaction and collaboration.	6.6	6.5
Face-to-face activities contributed meaningfully toward achieving the course learning objectives.	6.5	6.4
Assessments (e.g., tests and self-assessments) were appropriate for the course objectives, content, and activities.	5.4	5.5
I provided adequate feedback on residents’ learning progress.	5.7	5.9
Learner assessments and provision of feedback proceeded smoothly.	5.4	5.9
The course will be easy to maintain and deliver again.	6.0	6.2
I had access to needed tools during course delivery.	5.8	6.2
I received adequate support for any technical issues encountered while developing and delivering this course.	6.2	6.3
I was able to provide adequate support to students for questions or concerns about their learning.	6.0	6.2
The course was a good use of time and resources.	6.4	6.3
I was satisfied with the overall quality of the course.	6.5	6.5

## Discussion

This paper addresses a current and growing challenge in postgraduate medical education. Namely, the development and distribution of competency-based curricula designed to teach and assess EPAs that are not well suited to a clinical-based model. Moreover, CBME requires that postgraduate training programs directly observe the abilities of their trainees and provide them with formative feedback, even for rare and unpredictable EPAs. Developing simulation-based curricula to address these challenges will require significant resources. Instead of creating simulation curricula de novo within each training program or institution, we believe that certain EPAs and their instructional and assessment methods are well-suited for widespread distribution.

In addition to the efficiency rationale for using established curricula and their programs of assessment, there is a tremendous potential for scholarship. The small sample size is very often a limitation of educational research. The use of similar curricula and assessment tools at different institutions allows for a more robust synthesis of data for research purposes. The implementation of a common curriculum at a second institution has effectively doubled our subject numbers and will allow analyses that were not previously possible.

This paper describes the comprehensive and successful transfer of a resuscitation curriculum to a second institution. To facilitate further adoption of this curriculum, we plan to make it readily available online. The simulation cases used for the program will be peer-reviewed and published on EMSimCases.org using their standardized template. A blog post published on the site will provide an overview of the simulation program and allow the faculty from other institutions to access the curriculum and course-specific assessment and program evaluation tools.

The costs associated with this curriculum were $120 per session at Queen’s University and $96 per session at the University of Saskatchewan. This amount paid the nursing confederate and simulation technician salaries. Additional costs included faculty time and the use of the simulation laboratory. At Queen’s University, faculties receive “teaching points” for their respective departments and access to the simulation laboratory was granted through the Office of Postgraduate Medical Education. At the University of Saskatchewan, faculties are paid variable rates depending on their respective agreements with the College of Medicine and access to the simulation laboratory was funded via block payments to the simulation centers by the College of Medicine.

## Conclusions

We have demonstrated the feasibility of transposing a multi-disciplinary simulation-based resuscitation curriculum from one institution to another, including cases, assessment tools, and program evaluation instruments. We hope this example of successful curricular adaptation will encourage other institutions and departments to foster collaboration in future curriculum development and scholarship.

## Figures and Tables

**Figure 1:**
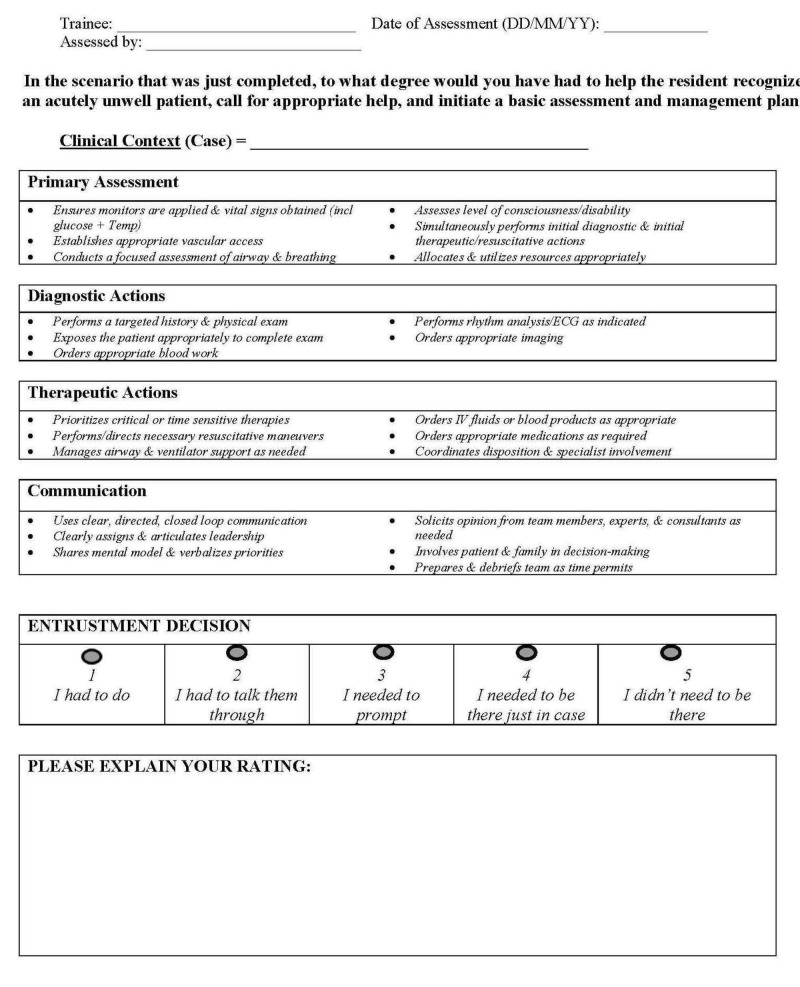
Generic Assessment Tool (Appendix 1)

**Figure 2:**
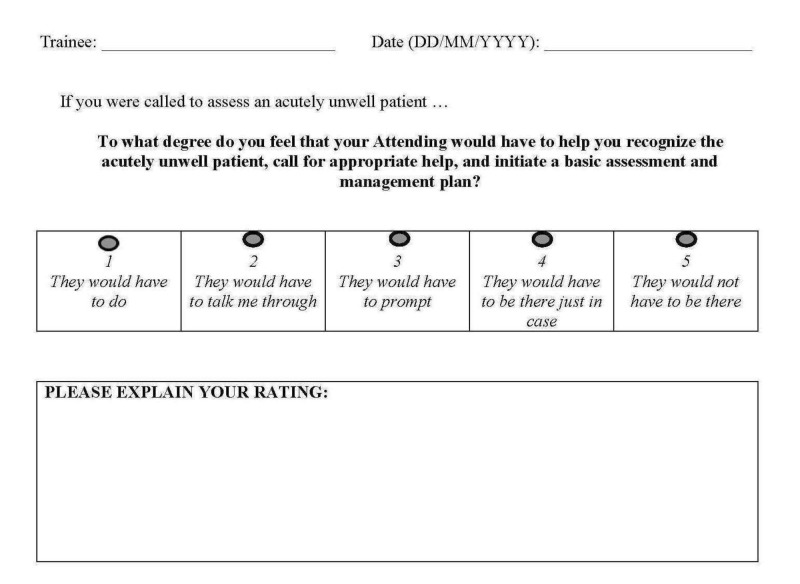
General Confidence Score (Appendix 2)

**Figure 3:**
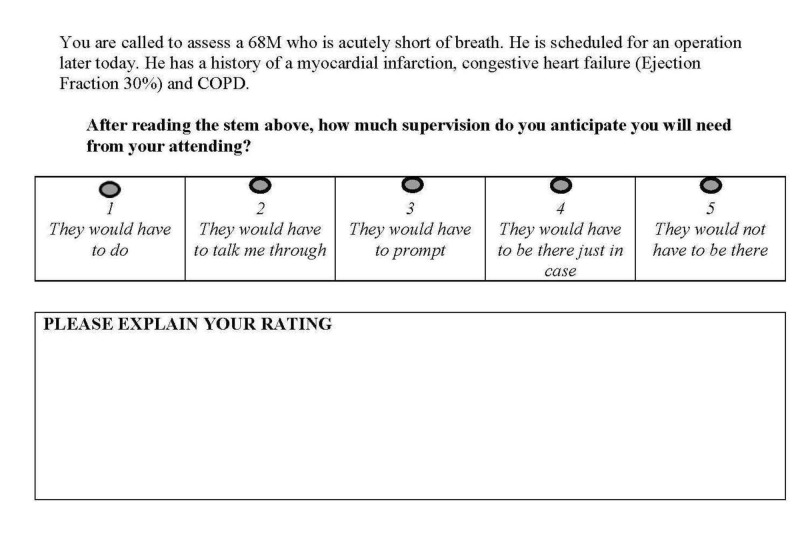
Case-specific Confidence Score Example (Appendix 3)
